# Supplementary material for: Effect of Hydrothermal Aging on Damping Properties in Sisal Mat-Reinforced Polyester Composites
Source: Polymers (Basel). 2024 Jan 5;16(2):166. doi: 10.3390/polym16020166 (PMC10818767; doi:10.3390/polym16020166)
Supplement: Supplementary file 1 [file polymers-16-00166-s001.zip › polymers-2603862-supplementary.pdf]

polymers

Article

Effect of Hydrothermal Aging on Damping Properties in Sisal Mat-Reinforced Polyester Composites

Moisés Silva [1,2,*], Beatriz Silva [1], Adrielle Marques [1], Silvana Mattedi [3,*], Ricardo Carvalho [1]

[1] Construction and Structures Department –Federal University of Bahia, Salvador, BA, Brazil; ricardoc@ufba.br
[2] SENAI Institute of Technology in Civil Construction, SENAI CIMATEC, Salvador, 41650010, BA, Brazil; moises.silva@fieb.org.br
[3] Chemical Engineering Graduate Program –Federal University of Bahia, Salvador, BA, Brazil; silvana@ufba.br
* Correspondence: RC.: ricardoc@ufba.br, SM: silvana@ufba.br; Tel.: 557142839813,

Supplementary Material

Tables S1 to S3 show sisal fibers physical, mechanical and chemical properties from literature

Table S1. Physical properties of sisal fibers. Adapted from: (Peças et. al.,[1])

**Physical Properties**	**Value**
Density (g/cm³)	1.36
Diameter (μm)	7-47
Moisture Content (%)	11

Table S2. Mechanical properties of sisal fibers. (Peças et al., [1])

**Mechanical Properties**	**Value**
Tensile (MPa)	577-855
Young's Modulus (GPa)	9-22
Elongation at Break (%)	1.9-3

Table S3. Chemical Properties of sisal fibers. (Gurunathan et al., [2])

**Chemical Properties**	**Value**
Cellulose (wt%)	67-78
Hemicellulose (wt%)	10-14
Lignin (wt%)	8-11
Pectin (wt%)	10
Water soluble (wt%)	1.3
Wax (wt%)	2
Microfibrillar angle (deg.)	10-22

References

1. Peças, P., H. Carvalho, H. Salman, and M. Leite. (2018). "Natural Fibre Composites and Their Applications: A Review." Journal of Composites Science 2 (4): 66. doi:10.3390/jcs2040066.

2. Gurunathan T., S. Mohanty, S. K. Nayak, (2015) "A review of the recent developments in biocomposites based on natural fibres and their application perspectives". Composites Part A: Applied Science and Manufacturing, 77, 1-25,doi:10.1016/j.compositesa.2015.06.007.

Disclaimer/Publisher's Note: The statements, opinions and data contained in all publications are solely those of the individual author(s) and contributor(s) and not of MDPI and/or the editor(s). MDPI and/or the editor(s) disclaim responsibility for any injury to people or property resulting from any ideas, methods, instructions or products referred to in the content.
